# Prehospital treatment of patients with acute intracranial pathology: adherence to guidelines and blood pressure recommendations by the Danish Air Ambulance

**DOI:** 10.1186/s13049-018-0534-x

**Published:** 2018-08-22

**Authors:** Joachim Juelsgaard, Leif Rognås, Lars Knudsen, Troels Martin Hansen, Mads Rasmussen

**Affiliations:** 1 The Danish Air Ambulance, Central Denmark Region, Aarhus, Denmark; 2 The Prehospital Critical Care Service, Central Denmark Region, Aarhus, Denmark; 3 Department of Anaesthesia and Intensive Care, Section of Neuroanaesthesia, Aarhus University Hospital, Aarhus, Denmark

**Keywords:** Helicopter emergency medical service, Guideline adherence, Cerebral pathology, Traumatic brain injury

## Abstract

**Background:**

Hypoxia and hypotension may be associated with secondary brain injury and negative outcomes in patients with traumatic and non-traumatic intracranial pathology. Guidelines exist only for the prehospital management of patients with severe traumatic brain injury (TBI). In patients with non-traumatic intracranial pathology, TBI guideline recommendations may be applied to assess whether hypoxia and hypotension should be avoided during prehospital treatment. The main study objective was to assess the extent to which Danish Helicopter Emergency Medical Service (HEMS) critical care teams adhere to the prehospital TBI guideline recommendations for the management of patients with a clinical diagnosis of non-traumatic intracranial pathology or isolated TBI. Furthermore, in the same two groups of patients, we evaluated the adherence of the Danish HEMS critical care teams to recommendations aiming to maintain systolic blood pressure (SBP) > 110 mmHg and > 120 mmHg.

**Methods:**

In total, 211 prehospital patient records were studied. All patients were treated for non-traumatic intracranial pathology or isolated TBI by the Danish HEMS critical care teams from October 1, 2014, to January 1, 2017. Adherence to the prehospital TBI guideline recommendations and the SBP recommendations above was assessed in non-TBI and TBI populations.

**Results:**

The adherence rates to TBI guideline recommendations among Danish HEMS critical care teams were 69% (*n* = 106 [95% CI: 61–77%]) in the non-TBI population and 74% (*n* = 43 [95% CI: 61–85%]) in the TBI population. SBP > 110 mmHg was observed in 74% (*n* = 113 [95% CI: 66–81%]) and 69% (*n* = 40 [95% CI: 56–81%]) of cases in the non-TBI and TBI population, respectively. SBP > 120 mmHg was observed in 55% (*n* = 84, [95% CI: 47–63%]) of patients in the non-TBI population and 55% (*n* = 32 [95% CI: 42–68%]) of the patients in the TBI population.

**Conclusions:**

Due to a lack of comparative data, it is difficult to determine the performance quality of the Danish HEMS critical care teams. Our findings may suggest that adherence to TBI guidelines and SBP recommendations needs to be a continuous focal point for the Danish HEMS to avoid secondary brain damage.

## Background

Acute cerebral pathology can be due to severe traumatic brain injury (TBI) or stroke, which are potential catastrophic injuries associated with impaired quality of life and high mortality [1, 2]. Studies in patients with TBI have demonstrated that even brief episodes of hypoxia and hypotension may be associated with secondary brain injury and negative outcomes [3, 4]. The same may apply to patients with acute non-traumatic intracranial pathology; however, data on the physiological predictors of poor outcome are not available for this group of patients. Accordingly, prehospital treatment of airway obstruction, insufficient oxygenation and unstable haemodynamics is considered essential for a favourable outcome in both groups of patients [5–7]. Current guidelines for the prehospital management of TBI recommend prehospital endotracheal intubation in patients with a Glasgow Coma Score (GCS) < 9, a peripheral blood oxygen saturation (SpO2) maintained above 90%, and systolic blood pressure (SBP) greater than 90 mmHg [5, 7]. However, new evidence suggests that the threshold for hypotension-induced secondary brain injury in patients with TBI may be as high as an SBP of 110 mmHg [8] or 120 mmHg [3, 9]. Consequently, the current standard operating procedure (SOP) for the Danish Helicopter Emergency Medical Service (HEMS) recommends maintaining SBP > 110 mmHg in patients with isolated TBI [8]. However, no recommendations exist for the prehospital targets for SpO2 and SBP in patients with a clinical diagnosis of acute non-traumatic intracranial pathology. Both traumatic and non-traumatic brain injury share similar pathophysiological mechanisms, including increased intracranial pressure (ICP) and associated compromised cerebral perfusion and oxygenation. In this study, we therefore apply the TBI guideline recommendations above as a measure for the performance of Danish HEMS critical care teams in the prehospital treatment of patients with acute non-traumatic intracranial pathology. Currently, data are not available on the management of patients with suspected acute intracranial pathology by HEMS critical care teams. Thus, the primary objective of this observational study was to assess the extent to which the Danish HEMS critical care teams adhere to the prehospital TBI guideline recommendations for the treatment of patients with a clinical diagnosis of acute non-traumatic intracranial pathology or isolated TBI [5]. As secondary aims, we assessed the extent to which Danish HEMS critical care teams adhere to more recent blood pressure recommendations of an SBP > 110 mmHg [8] and > 120 mmHg [3, 9] in the prehospital management of patients with a clinical diagnosis of non-traumatic intracranial pathology or isolated TBI.

## Methods

### Study design and study period

This retrospective study included records from patients treated by the Danish HEMS from October 1, 2014, to January 1, 2017.

### Setting

The Danish HEMS is an integral part of the government funded Danish Emergency Medical Service (EMS) and provides prehospital critical care service to all 5.7 million Danish citizens. The Danish EMS and prehospital critical care service has previously been described in detail [10, 11]. From three bases across Denmark, the Danish HEMS organization operates three helicopters covering an area of 42.394 km^2^. Similar to other HEMS systems in Scandinavia and Central Europe, Danish HEMS helicopters are staffed with a pilot, a paramedic (HEMS crew member) and a consultant anaesthesiologist with advanced prehospital and critical care management training and experience. The Danish HEMS service is dispatched by five regional Emergency Medical Coordination Centres (EMCC) according to a criteria-based dispatch protocol [12].

### Patients

This study included patients with a GCS < 9 and a clinical diagnosis of either non-traumatic intracranial pathology or isolated TBI. Patients of all ages and both genders were included. The study is based on prehospital clinical diagnoses made by the prehospital critical care physician on scene. These included diagnoses correlated with isolated TBI (i.e., diffuse traumatic brain lesion, traumatic intracranial haemorrhage, and traumatic skull fracture) and non-traumatic diagnoses, including ischaemic stroke, subarachnoid haemorrhage, and intracerebral haemorrhage. Records in the database with clinical diagnoses of meningitis and epileptic seizures were also reviewed because these diagnoses may occasionally disguise severe non-traumatic cerebral pathology.

### Data

Data were extracted from the Danish HEMS database, in which all prehospital missions are registered, including prehospital diagnoses. The database provides information on patient identification and patient demographics, including treatment. In each specific case with suspected acute intracranial pathology, the HEMS anaesthesiologist recorded patient identification data (social security number) and physiological parameters, including the blood oxygen saturation, blood pressure, heart rate and treatment, on a specific paper patient chart. The number of physiological recordings varied. Subsequently, data concerning patient identification, patient demographics and treatment, including prehospital diagnosis, were entered into the Danish HEMS database. The HEMS patient charts were stored in a secure locker at the prehospital compound. Following extraction of data with prehospital diagnoses from the HEMS database, corresponding patient charts were reviewed to record physiological parameters and determine adherence to guidelines and SBP recommendations.

### Endpoints

- Prevalence of adherence to the TBI guideline recommendations, which include endotracheal intubation in patients with a GCS < 9, SpO2 > 90% and SBP > 90 mmHg [5].
- Prevalence of SBP recordings per guideline (SBP > 90 mmHg [5]) and recommended SBP levels (SBP > 110 mmHg [8] and SBP > 120 mmHg [3]) at any time during prehospital HEMS care.
- Prevalence of SpO2 recordings according to guideline recommendation (> 90%) at any time during prehospital HEMS care [5].

### Statistics

Proportions are reported with 95% confidence intervals (CI). Age is reported as the mean (range).

### Ethics

According to Danish law, permission from the ethics committee or from individual patients is not required for register-based studies. The Danish HEMS database is approved by the Danish Data Protection Agency.

## Results

**Fig.1:**
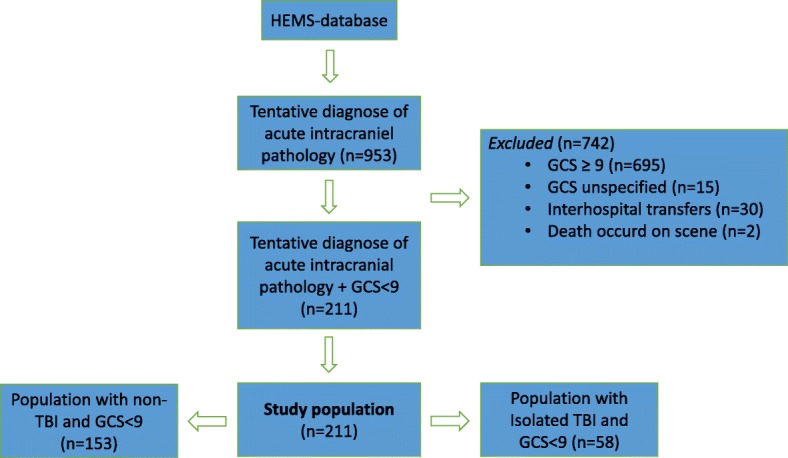
Flow chart of the study population generated from the HEMS database. TBI: traumatic brain injury

During the study period, a total of 3278 patients were treated by the Danish HEMS critical care teams and transported by either HEMS or ground ambulance (activity data provided from the steering committee of the Danish HEMS (Fig. 1). Overall, 953 subjects met the inclusion criterion of suspected acute intracranial pathology. In total, 695 patients had a GCS ≥ 9 and were excluded from the study. Thirty patients were excluded because the air ambulance provided inter-hospital transfer service only in these cases. An additional 15 subjects were excluded due to missing patient charts. The final study population consisted of 211 patients with a GCS < 9 and a prehospital diagnosis of acute intracranial pathology. In total, 153 patients were diagnosed with acute non-traumatic intracranial pathology (non-TBI), and 58 patients were diagnosed with isolated TBI. Population demographics are presented in Table 1. A median of three SpO2% recordings and three SBP measurements were recorded for each patient.

**Table 1:** Demographics of the populations with non-traumatic intracranial pathology (non-TBI population) and isolated TBI (TBI population)

	Non-TBI population	TBI population
Number of patients (n)	153	58
Age [mean (range)] (years)	60 (2–92)	42 (0–87)
< 16 years, n (%)	8 (5.2)	10 (17.2)
< 2 years, n (%)	0 (0)	2 (3.4)
Unspecified age, n (%)	1 (0.6)	1 (1.7)

### Adherence to guidelines and SBP recommendations

#### Adherence to TBI guideline recommendations

The adherence of HEMS physicians to TBI guidelines and SBP recommendations is presented in Table 2. Guideline recommendations (endotracheal intubation, SpO2 > 90% and SBP > 90 mmHg) were fulfilled in 69% (*n* = 106 [95% CI: 61–77%]) of the cases in the non-TBI population and 74% (*n* = 43 [95% CI: 61–85%]) of the cases in the isolated TBI population.

**Table 2:** Adherence to TBI guidelines and SBP recommendations in the population of patients with non-traumatic intracranial pathology (non-TBI population) and the population with isolated TBI (TBI population)

Physiologic guideline references	Non-TBI population n (%, [95% CI])	TBI population n (%, [95% CI])
Number of patients	153	58
Adherence to TBI guideline recommendations (intubation when GCS < 9, SpO2% > 90 + SBP > 90 mmHg)	106 (69%, [61–77%])	43 (74%, [61–85%])
SBP > 90 mmHg	134 (88%, [81–92%])	53 (91%, [81–97%])
SBP > 110 mmHg	113 (74%, [66–81%])	40 (69%, [56–81%])
SBP > 120 mmHg	84 (55%, [47–63%])	32 (55%, [42–68%])
SpO2% > 90	147 (96%, [92–99%])	50 (86%, [75–94%])

*SBP* systolic blood pressure, *SpO2* peripheral blood oxygen saturation

#### Blood pressure

Adherence to the SBP guideline recommendations (SBP > 90 mmHg) and SBP recommendations (SBP > 110 and > 120 mmHg) are presented in Table 2. In the non-TBI population, recordings of SBP > 90, SBP > 110 and SBP > 120 mmHg were identified in 88% [95% CI: 81–92%], 74% [95% CI: 66–81%] and 55% [95% CI: 47–63%] of the cases, respectively.

In the TBI population, recordings of SBP > 90, SBP > 110 and SBP > 120 mmHg were found in 91% [95% CI: 81–97%], 69% [95% CI: 56–81%] and 55% [95% CI: 42–68%] of the cases, respectively.

#### Oxygen saturation

Adherence to the SpO2 guideline threshold is presented in Table 2. In the non-TBI population, 6 patients (4%) had SpO2 values < 90%. In the TBI population, 8 patients (14%) exhibited oxygen saturation values < 90%. With the exception of one case, all patients with SpO2 values below the guideline threshold recommendation were intubated.

## Discussion

Complete adherence to the prehospital TBI guideline recommendations (endotracheal intubation when GCS < 9, SpO2 > 90% and SBP > 90 mmHg) was observed in 69% of the cases in the non-TBI population and 74% of the cases in the TBI population. Only 74% of the non-TBI population and 69% of patients with isolated TBI were managed according to the blood pressure SOP of the Danish Air Ambulance, which recommends SBP > 110 mmHg. According to the best of our knowledge, no previous data exist reporting the adherence to TBI guideline recommendations in a population of patients with a clinical diagnosis of non-traumatic intracranial pathology. Consequently, it is difficult to determine the quality of performance by the Danish HEMS critical care teams. However, the reported adherence to guideline recommendations indicates that there is room for improvement.

The Danish HEMS critical care teams maintained SBP > 90 mmHg in 88% of non-TBI cases and 91% of TBI cases. This rate is high compared to a previous study in TBI patients treated by Danish ground-based anaesthesiologist-staffed prehospital critical care teams, in which 82% of the patients had an SBP > 90 mmHg [10]. In the previous study by Rognås et al., SBP was measured immediately after intubation, which may explain the difference in the prevalence of hypotension between the two studies [10]. The increased guideline adherence by HEMS critical care teams may also be explained by their extensive prehospital experience.

The European Brain Injury Consortium suggests that non-adherence to the recommendation of maintaining the SBP > 120 mmHg is associated with secondary brain damage in patients with TBI [3, 9]. In this study, only 55% of patients in both the non-TBI population and the TBI subpopulation maintained an SBP greater than 120 mmHg. This observation is consistent with the study by Rognås et al., which reported a 51% prevalence of SBP > 120 mmHg in patients with TBI [10]. The high prevalence of SBP values less than 120 mmHg may be explained by the fact that most of these patients were intubated and sedated. Thus, although it is unknown whether the high prevalence of SBP < 120 mmHg was associated with a poor outcome, this finding emphasizes the importance of prehospital critical care teams focusing on haemodynamic management during the prehospital phase.

The prevalence of oxygen saturation > 90% was 96% in the non-TBI population and 86% in TBI patients. The slightly greater number of hypoxic cases in the TBI subpopulation could reflect brain impact apnoea or additional pulmonary injuries not initially diagnosed by the HEMS physician. A previous Italian HEMS study investigating TBI patients and hypoxia prior to intubation revealed an increase in mortality of 27–50% when desaturation occurred [13], emphasizing the importance of adhering to these recommendations. The low prevalence of hypoxia in this study is likely explained by the high adherence to current airway management guidelines that recommend intubation and artificial ventilation in patients with TBI and a GCS < 9 [7, 10, 11]. This practice is further applied by Danish HEMS physicians in airway management of patients with non-traumatic acute intracranial pathology. Accordingly, the airway guideline adherence rate in this study was 84% in the non-TBI population and 93% in TBI patients. This observation is similar to the findings by Rognås et al., who reported 93% guideline adherence by EMS physicians in their prehospital airway management of patients with TBI [10]. Furthermore, a recent study demonstrated that a physician-staffed HEMS is associated with a reduced prevalence of prehospital hypoxia in TBI patients [14]. The similar findings across Danish prehospital services reflect a consistent high quality with regard to guideline adherence in prehospital airway management. This finding implies that Danish prehospital physicians are performing at similar standards in both ground-based and HEMS services.

### Study limitations

This study was a retrospective study, and the following study limitations should be considered in the interpretation of the study results. The relatively small sample size may not be representative of a large population consisting of patients with traumatic and non-traumatic brain injury and should be considered in the interpretation of the results. Currently, no prehospital guidelines or recommendations exist regarding respiratory and haemodynamic management in patients with non-traumatic acute intracranial pathology and a GCS < 9. Thus, whether the abovementioned guideline recommendations apply to this group of patients is currently unknown and may have influenced the HEMS physicians’ decisions and prehospital treatment. The prehospital continuous physiological parameters were all registered on a chart by the HEMS physician and cannot be independently verified. In addition, the first physiological measurements recorded on scene were often performed by ground-based ambulance personal, and treatment to stabilize the patient was often initiated before arrival of the Danish HEMS. Interpersonal variation in the number of physiological recordings and the time interval between each recording is another limitation. Consequently, patients initially classified as having physiological values greater than the guideline and SBP recommendations may have experienced periods with physiological readings below the guideline and recommended levels. Outcome data were not reported in this study, and it is unknown whether adherence to guidelines and blood pressure recommendations improved patient outcomes. Randomized studies are needed to examine the association between guideline/recommended physiological values and outcomes.

## Conclusions

The self-reported rate of adherence to TBI guideline recommendations (endotracheal intubation when the GCS < 9, SBP > 90 mmHg and SpO2 > 90%) among Danish HEMS physicians was 69% in the non-TBI population and 74% in patients with isolated TBI. Our findings may suggest that adherence to guidelines and SBP recommendations to avoid secondary brain damage should be a continuous focal point for Danish HEMS critical care teams.

Randomized studies are needed to examine the association between guideline/recommended physiological values and outcomes.

## Abbreviations

EMCC: Emergency Medical Coordination Centre
EMS: Emergency medical service
GCS: Glasgow coma score
HEMS: Helicopter Emergency Medical Service
SBP: Systolic blood pressure
SOP: Standard operating procedure
SpO2: Peripheral blood oxygen saturation
TBI: Traumatic brain injury
